# MRGPRX2 signaling involves the Lysyl-tRNA synthetase and MITF pathway

**DOI:** 10.3389/fimmu.2023.1154108

**Published:** 2023-05-10

**Authors:** Yanru Guo, Laia Ollé, Elizabeth Proaño-Pérez, Cristina Aparicio, Mario Guerrero, Rosa Muñoz-Cano, Margarita Martín

**Affiliations:** ^1^ Biochemistry and Molecular Biology Unit, Biomedicine Department, Faculty of Medicine and Health Sciences, University of Barcelona, Barcelona, Spain; ^2^ Clinical and Experimental Respiratory Immunoallergy (IRCE), Institut d’Investigacions Biomediques August Pi i Sunyer (IDIBAPS), Barcelona, Spain; ^3^ Faculty of Health Sciences, Technical University of Ambato, Ambato, Ecuador; ^4^ Allergy Department, Hospital Clinic, University of Barcelona, Barcelona, Spain; ^5^ Redes de Investigación Cooperativa Orientadas a Resultados en Salud (RICORS), Instituto de Salud Carlos III, Madrid, Spain

**Keywords:** MRGPRX2, LysRS, MITF, mast cell degranulation, adverse drug reactions

## Abstract

MRGPRX2, a G-protein-coupled-seven transmembrane domain receptor, is mainly expressed in mast cells and neurons and is involved in skin immunity and pain. It is implicated in the pathophysiology of non-IgE-mediated immediate hypersensitivity and has been related to adverse drug reactions. Moreover, a role has been proposed in asthma, atopic dermatitis, contact dermatitis, and chronic spontaneous urticaria. Although it has a prominent role in disease, its signaling transduction is poorly understood. This study shows that MRGPRX2 activation with substance P increased Lysyl t-RNA synthetase (LysRS) translocation to the nucleus. LysRS is a moonlighting protein with a dual role in protein translation and IgE signaling in mast cells. Upon allergen- IgE-FcεRI crosslinking, LysRS is translocated to the nucleus and activates microphthalmia-associated transcription factor (MITF) activity. In this study, we found that MRGPRX2 triggering led to MITF phosphorylation and increased MITF activity. Therefore, overexpression of LysRS increased MITF activity after MRGPRX2 activation. MITF silencing reduced MRGPRX2-dependent calcium influx and mast cell degranulation. Furthermore, a MITF pathway inhibitor, ML329, impaired MITF expression, calcium influx, and mast cell degranulation. Moreover, drugs such as atracurium, vancomycin, and morphine, reported to induce MRGPRX2-dependent degranulation, increased MITF activity. Altogether, our data show that MRGPRX2 signaling enhances MITF activity, and its abrogation by silencing or inhibition resulted in defective MRGPRX2 degranulation. We conclude that MRGPRX2 signaling involves the LysRS and MITF pathway. Thus, MITF and MITF-dependent targets may be considered therapeutic approaches to treat pathologies where MRGPRX2 is implicated.

## Introduction

Mast-related G-protein-coupled receptor member X2 (MRGPRX2) is mainly expressed on mast cells (MC) (abundantly in skin MC) and neurons. MRGPRX2 is physiologically involved in host defense, tissue homeostasis and repair, nociception, inflammatory pain, and itch (1, 2). Endogenous and exogenous ligands have been described to bind MRGPRX2. Endogenous ligands such as substance P, human β-defensins, or vasointestinal peptide, directly involved in pain and itch, are directly related to its physiological functions (3). These effects can become chronic inflammation in a pathological state (4–6). Regarding exogenous ligands, several have been identified, such as mastoparan (bee wasp venom component), cationic peptidergic drugs, neuromuscular blocking agents (NMBAs) (e.g., atracurium, cisatracurium, and rocuronium), opiates, and antibiotics such as fluoroquinolones and vancomycin. Most of these exogenous ligands are the molecular basis of MRGPRX2-dependent adverse drug reactions (7, 8). Several molecules, such as natural compounds, cytokines, peptides, and DNA aptamers, have been described to prevent MRGPRX2-mediated pseudo-allergic responses and inflammation (9–11).

Yet, MRGPRX2 signaling is still poorly understood. To better understand how to modulate MRGPRX2 actions to dodge pathological states, some progress in unrevealing MRGPRX2 signaling has been made; however, more effort is needed. Proteins G, Gαi, and/or Gαq are involved in the early signals (12, 13), and calcium mobilization is rapid and transient (14). Further downstream events include MAP kinases (ERK1/2), p38, and PI3K (15). Furthermore, GTPase activation (Cdc42) upstream of the unconventional class I myosin 1f (MYO1F) regulates actin cytoskeleton dynamics and granule release (16). Lately, β arrestins 1 and 2 (involved in GPCR desensitization) have been proposed to interfere with MRGPRX2-dependent degranulation (17).

Microphthalmia associated-transcription factor (MITF) is a basic-helix-loop-helix-leucine zipper (bHLH-ZIP) family member. MITF involves many biological processes, including cell differentiation, survival, senescence, metabolism, and DNA damage repair (18). The principal cell types affected in MITF-deficient mice are MCs, osteoclasts, and melanocytes (19). Moreover, MC and basophil cell fates are determined by the antagonistic regulation of C/EBPα and MITF (20). Additionally, MITF is downstream of KIT and FcεRI pathways (21–23). Nevertheless, most information about MITF stems from its essential role in melanocyte biology and melanoma as an oncogene (24–26). Post-translational modifications are primarily subject to the transcriptional activity of MITF. MITF is phosphorylated by ERK1/2 at Ser73 and by p90 ribosomal S6 kinase (p90RSK) at Ser409 (27–29). MITF phosphorylation at Ser73 has been associated with increased activity and double phosphorylation at Ser73 and Ser409 has been related to proteasome degradation (29). MITF activity can be repressed by HINT1 (histidine triad nucleotide-binding protein 1) (30).

Lysyl-tRNA synthetase (LysRS), a paradigm of a multifunctional protein in MCs, is involved in protein translation (canonical function) and upon FcεRI triggering in MITF activation (non-canonical role) (23). After IgE crosslinking, MAP kinase activation phosphorylates cytosolic LysRS (Ser 207). Phosphorylation in LysRS alters its binding to the multi-tRNA synthetase complex (MSC)-p38 in the cytosol and allows translocation to the nucleus, synthesizing diadenosine tetraphosphate (Ap4A). The Ap4A accumulated in the nuclei of IgE-activated MCs binds to HINT1, a MITF repressor, and liberates MITF, activating MITF-dependent gene expression. MITF regulates numerous genes encoding essential proteins involved in MC proinflammatory events, such as histidine decarboxylase (Hdc) (31), which catalyzes histamine synthesis; granzyme B (GrB), which participates in the cytotoxic action of MCs (32) or PGD2 synthase increasing PGD2 levels (33).

Our group recently characterized the mutation LysRS P542R in a patient with severe anaphylaxis to Hymenoptera. A substitution of proline by arginine disrupts the protein’s functional motion, promoting an open state similar to the phosphorylated wild-type form. Altogether, this results in a constitutive increase in MITF activity (34).

MRGPRX2 has been related to several pathologies, from immediate-type hypersensitivity reactions (adverse drug reactions, Hymenoptera venoms reactions) to prolonged type- 2 inflammation (such as chronic asthma or chronic spontaneous urticaria) (1, 2, 4). Therefore, it is essential to understand the molecular groundwork of MRGPRX2 and the associated intracellular signaling components to manage the treatment of MRGPRX2-associated diseases. The current study explores the MRGPRX2-LysR-MITF pathway in mast cell exocytosis.

## Materials and methods

### Antibodies and reagents

Anti-MITF (clone D5G7V) and phospho-p44/42 MAPK antibodies were from Cell Signaling Technology (Danvers, MA, USA). Anti-phospho-MITF (Ser73/180) and mouse anti-αTubulin antibodies were from Sigma-Aldrich (St. Louis, MO, USA). Rabbit anti-Lamin β1 antibody was purchased from Abcam (Cambridge, UK). PE anti-human FcϵRI antibody was obtained from Thermo Fisher Scientific (Waltham, MA, USA). Anti-LysRS (D-4) and PE anti-human CD117 were obtained from Santa Cruz Biotechnology, Inc (Santa Cruz, CA, USA). PE anti-human MRGPRX2 was from Bio Legend (San Diego, CA, USA ). APC anti-human CD63 and FITC Annexin V were from ImmunoTools GmbH (Friesoythe, Germany). Biotinylated human IgE was from Abbiotec (San Diego, CA, USA), and streptavidin was from Sigma (St. Louis, MO, USA). Substance P was from AnaSpec (Fremont, CA). ML329 was obtained from Axon Med Chem (Groningen, The Netherlands). Morphine was obtained from B. Braun Medical S.A (Spain), the muscle relaxant atracurium was from Pfizer Inc (NY, USA), meglumine amidotrizoate was from Juste Laboratories (Spain), and the vancomycin antibiotic was from Normon (Spain).

### Cell culture

The LAD2 human mast cell line was a kind gift from Drs. A. Kirshenbaum and D.D. Metcalfe (National Institutes of Health, Bethesda, MD), culturing in StemPro-34 media, supplemented with StemPro-34 nutrient (Thermo Fisher Scientific; Waltham, MA, USA) and 2mM L-glutamine (Lonza), 100 U/mL penicillin (Lonza) and 100 μg/mL streptomycin (Lonza), and 100 ng/mL SCF (ImmunoTools GmbH, Friesoythe, Germany) (35). Primary human mast cells (huMCs) derived from CD34^+^ -positive peripheral blood cells were obtained from healthy donors, CD117 MicroBeads Kit (Mitenyi Biotec, Germany) for CD34^+^ progenitor cell isolation. Cells were cultured for 0-2 weeks with 100 ng/ml SCF, IL-6, and IL-3 (ImmunoTools GmbH, Friesoythe, Germany) and 2-6 weeks with 100 ng/ml IL-6 and SCF. After six weeks, CD34^+^ -derived human MCs were assessed by surface expression of FcϵRI and CD117. The expression of anti-human PE-MRGPRX2 was also checked for experiments. The HEK 293LTV cell line (Cell Biolabs Inc, San Diego, CA, USA) was used for lentivirus production.

### Western blotting

7·10^6^ cells were activated with substance P (MedChemExpress, NJ, USA) at different times. Cellular fractioning was performed as described elsewhere (36). The protein concentration was determined using the Protein Assay Dye Bio-Rad Kit (Bio-Rad Laboratories, Inc. USA) according to the manufacturer’s recommendations. Electrophoresis was performed using NuPage TM 4-12% Bis-Tris Gel, 1.5 mm 15 w (Invitrogen, USA), and electrotransferred to polyvinylidene difluoride (PVDF) membranes (Millipore, Bedford, MA, USA). In all blots, proteins, after specific antibody incubation, were visualized by enhanced chemiluminescence (Western Bright TM ECL, Advansta, USA).

### Lentiviral transduction

Lentiviral particles were generated in HEK293LTV. The non-target (NT) sequence was as follows: 5’CCGGCAACAAGATGAAGAGCACCAACTCGAGTTGGTGCTCTTCATCTTGTTG TTTTT 3’, MITF shRNA 2 sequence: 5’CCGGCGGGAAACTTGATTGATCTTTCTCGAGAAAGATCAATCAAGTTTCCCGTTTTTG 3’. MITF shRNA 3 sequence: 5’ CCGGGGGAGCTCACAGCGTGTATTTCTCGAGAAATACACGCTGTGAGCTCCCTTTTTG 3’.

According to the manufacturer’s instructions, lentiviral particles to silence MITF gene expression were generated using Mission ^®^ shRNA technology (Sigma, St. Louis, MO, USA). LAD2 cells were transduced with polybrene 8 µg/ml (Santa Cruz) and selected with puromycin 1µg/ml. Cells were maintained with puromycin until the day of the experiment, when it was removed prior to cell activation.

### FACS staining

FcϵRI and MRGPRX2 expression were detected by direct staining with the indicated Abs for 30 minutes at 4°C. KIT expression was detected using PE- anti-human CD117. Cells were analyzed using a FACSCalibur flow cytometer (FACScan; BD Biosciences). Dead cells were excluded based on their Forward (FSC) and side scattering (SSC) profiles.

### Degranulation assays

Degranulation was analyzed based on CD63 expression on the cell membrane assessed by flow cytometry or via levels of β-hexosaminidase activity in the supernatant, as described in previous studies (8). Briefly, for CD63 determination: 1·10^5^ cells/point were incubated with Tyrode’s buffer or SP at 37°C for 30 min. Cells were incubated with blocking buffer (0.1% NaN_3_, 2% FBS, 20% rabbit serum, PBS) for 30 min on ice, and APC anti-human CD63 staining was performed for 30 min on ice. After washing, samples were incubated with propidium iodide (PI), acquired with a FACSCalibur flow cytometer, and analyzed with FlowJo software. PI-positive cells were excluded from the analysis. For β-hexosaminidase assays (37), **c**ells were seeded at 3·10^4^ cells/well in 96 well plates in Tyrode’s buffer and then activated with SP or left untreated for 30 min. An equal number of viable cells were used in each case. Supernatants were collected and incubated with P-nitrophenyl-N-acetyl-β-D-glucopyranoside (Sigma Aldrich) for 1h at 37°C. Triton (1%) was used to obtain total cell lysates of β-hexosaminidase. Absorbance was read at 405nm. β-hexosaminidase enzyme activity was expressed as the release percentage: β-hexosaminidase release = [cell degranulation/(cell degranulation + total lysate)] x100.

### PGD2 ELISA

PGD2 release was determined from the supernatants of cells (2.5·10^4^ cells/well) activated with substance P (2µM) overnight using a specific competitive Enzyme Immunoassay for PGD2 (Cusabio Technology LLC, Houston, TX, USA) according to the manufacturer’s instructions.

### Apoptosis assay

Apoptosis was measured using FITC Annexin V (BD Pharmingen, San Jose, CA, USA) according to the manufacturer’s suggested protocol and analyzed by flow cytometry. Caspase activity was assayed using the Caspases-Glo^®^ 3/7 Assay (Promega, San Luis Obispo, CA, USA) according to the manufacturer’s instructions.

### Calcium release assay

Cells were incubated with 2 µM Fluo-4, AM cell permeant (Thermo Fisher Scientific; Waltham, MA, USA), at 37°C for 30 minutes, then washed, and resuspended in Tyrode’s buffer. An equal number of viable cells were used in each case. After defining basal conditions, SP (or ionomycin) was immediately added to the cells (Sigma-Aldrich, St. Louis, MO, USA), and fluorescence was determined every 10 sec using a TECAN SPARK microplate reader.

### Luciferase assay

Firefly luciferase under the control of the TRPM1 promoter and control vector PGL3-Luciferase was a gift from David Fisher (Harvard Medical School) (38). 2.5·10^4^ cells were transfected with the Firefly and Renilla reporters at a ratio of 7:1, respectively. This reporter gene was transfected to the cells with a Renilla luciferase reporter gene, and the results were normalized to the empty vector-transfected conditions. Transfections were performed with lipofectamine. We used SP at a higher concentration for this assay to see significant gene reporter activation. Luciferase activity was detected using the Dual-Luciferase^®^ Reporter Assay System (Promega, Madison, WI) following the manufacturer’s instructions. Firefly Luciferase data were normalized according to Renilla luciferase data.

### GFP and LysRS WT-GFP transfection in LAD2 cells

LAD2 cells were transfected with human LysRS (KARS WT) pcDNA 3.1+C-eGFP vector and GFP empty vector (obtained from GenScript Biotech, The Netherlands)) as previously reported (34).

### Statistical analysis

Statistical analyses were performed using PRISM 9 (GraphPad Software, La Jolla, CA, USA). All results are expressed as mean ± standard error of the mean (SEM). After determining the normal distribution of the samples and variance analysis, a t-test was used to determine significant differences (p-value) between two experimental groups, and two-way ANOVA was used to determine significant differences (p-value) between several experimental groups. “*”P<0.05, “**” P<0.01, “***”P<0.001, “****”P<0.0001.

## Results

### MRGPRX2 activation increases the translocation of LysRS to the nucleus

**Figure 1:**
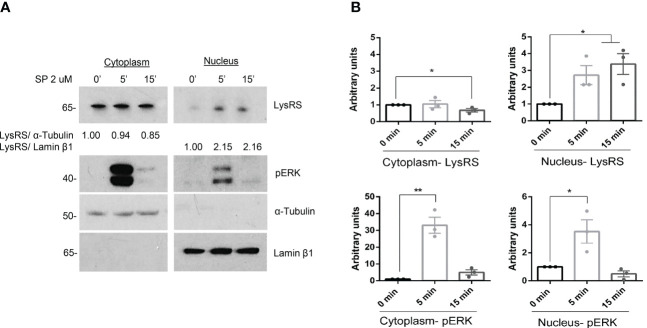
LysRS translocates to the nucleus after MRGPRX2 activation in mast cells (LAD2). **(A)** Cells were activated with 2 µM substance P (SP) at various times. Cytoplasmic and nuclear fractions were isolated, and western blots for LysRS and pERK were performed. α-tubulin and Lamin β1 markers for cytoplasm and nuclei, respectively, were used to assess cellular fraction purification and as loading controls. **(B)** The unpaired t-test was used for statistical analysis (*p < 0.05*, **p < 0.01). Experiments are the mean ± SEM (n=3).

MRGPRX2 signaling transduction increases ERK1/2 activity (15). On the other hand, Lysyl-tRNA synthetase (LysRS) is phosphorylated at Ser 207 and translocated into the nucleus downstream of IgE/FcϵRI signaling activation (23, 39). We wanted to explore whether MRGPRX2 signaling involves LysRS and its translocation to the nucleus. Ligands, such as SP, have shown MRGPRX2-dependent MC activity (40, 41). Thus, MCs (LAD2) were activated with substance P, cellular fractionation was carried out, and the LysRS location was explored. Our results show a rapid and transient phosphorylation of ERK1/2 and an increase in LysRS translocation to the nucleus of activated cells (**Figure 1**).

### MRGPRX2 activation increases MITF phosphorylation

**Figure 2:**
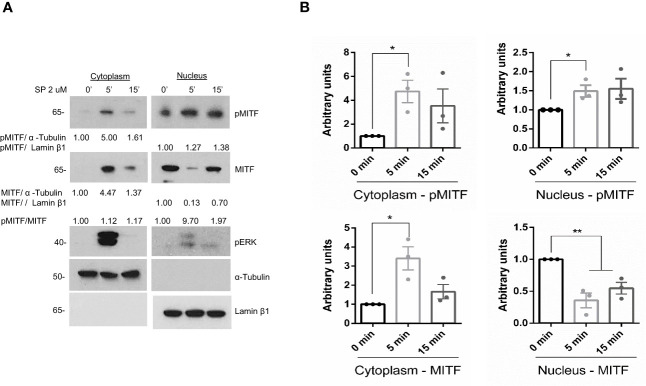
MITF is phosphorylated after MRGPRX2 activation in mast cells (LAD2). **(A)** Cells were activated with 2 µM substance P (SP) at various times. Cytoplasmic and nuclear fractions were isolated, western blots for pMITF (Ser 73/180), and MITF were performed, α-tubulin and Lamin β1 markers for cytoplasm and nuclei, respectively, were used to assess cellular fraction purification and as loading controls. **(B)** The unpaired t-test was used for statistical analysis (*p < 0.05, **p < 0.01). Experiments are the mean ± SEM (n=3).

Downstream of IgE signaling, the translocation of LysRS into the nucleus switches its catalytic activity in the production of diadenosine oligophosphate Ap4A, which binds to HINT, dissociating the MITF-HINT repressor complex and liberating MITF (23, 42). We next analyzed MITF activity in the context of MRGPRX2 activation. Since phosphorylation of MITF at Ser 73 has been associated with transcriptional activity (29), we examined whether MRGPRX2 increased MITF phosphorylation. As shown in **Figure 2**, MRGPRX2-dependent activation enhanced MITF phosphorylation in the cytoplasm and nucleus (note the pMITF/MITF ratio). Interestingly, MITF expression was mainly detectable in the nucleus before stimulation. However, after MRGPRX2 triggering, MITF levels increased in the cytosol. After 15 minutes, MITF returned close to the steady state. This is consistent with data regarding MITF posttranscriptional regulation in melanocytes where phosphorylation of MITF regulates its nuclear export, and via this export-import cycle, MITF activity can be tuned (43).

### MRGPRX2 signaling increases MITF activity

**Figure 3:**
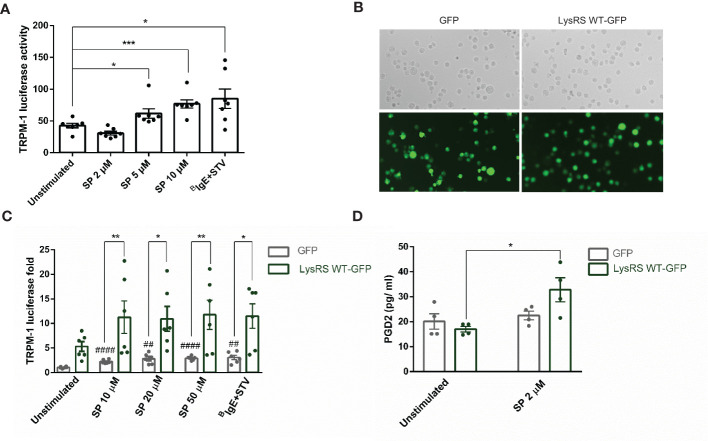
MRGPRX2 activation increases MITF activity via LysRS in mast cells (LAD2). **(A)** Cells were activated with 2 µM substance P (SP) at various concentrations for 6 hours in LAD2 cells transfected with TRPM1-luciferase gene reporter, as described in the materials and methods section. Biotinylated-IgE (0.1 µg/ml) was incubated overnight, and then, streptavidin (16 µg/mL) was added for 6 hours and used as a positive control. The unpaired t-test was used for statistical analysis (*p < 0.05, ***p < 0.001). **(B)** LAD2 cells were transfected with GFP and LysRS WT-GFP plasmids **(C)** and then activated at various SP concentrations, and biotinylated-IgE+ streptavidin was used as indicated above. The unpaired t-test used for statistical analysis (##p < 0.01, #### p<0.0001, SP *vs*. unstimulated GFP transfected LAD2 cells). The two-way ANOVA test was used for statistical analysis (*p < 0.05, **p < 0.01, GFP LAD2 *vs*. LysRS WT-GFP LAD2). **(D)** PGD2 after SP activation was measured in GFP and LysRS WT-GFP LAD2 cells. A two-way ANOVA test was used for statistical analysis (*p<0.05, GFP *vs*. LysRS WT-GFP). Experiments are the mean ± SEM (n=3).

Next, we assessed whether LysRS translocation to the nucleus and MITF phosphorylation after MRGPRX2 signaling resulted in increased MITF activity. We used a reporter gene assay, Melastatin 1 (TRPM1) promoter-controlled firefly luciferase, for that purpose. MITF is the main transcriptional regulator of TRPM1 (44). Thus, luciferase expression correlates with MITF activity (45). Results show that MITF activity increased after MRGPRX2 stimulation (**Figure 3A**). Moreover, when cells overexpressed LysRS WT-GFP (**Figure 3B**), a notorious increase in MITF activity was detected after substance P stimulation (**Figure 3C**). In that context, we also analyzed whether this increase in activity was concomitant to an increase in a MITF-dependent target such as PGD2 (33). Results show that PGD2 significantly increased in LysRS WT-GFP transfected cells after MRGPRX2 activation (**Figure 3D**).

### MITF silencing decreases MRGPRX2-dependent mast cell degranulation

**Figure 4:**
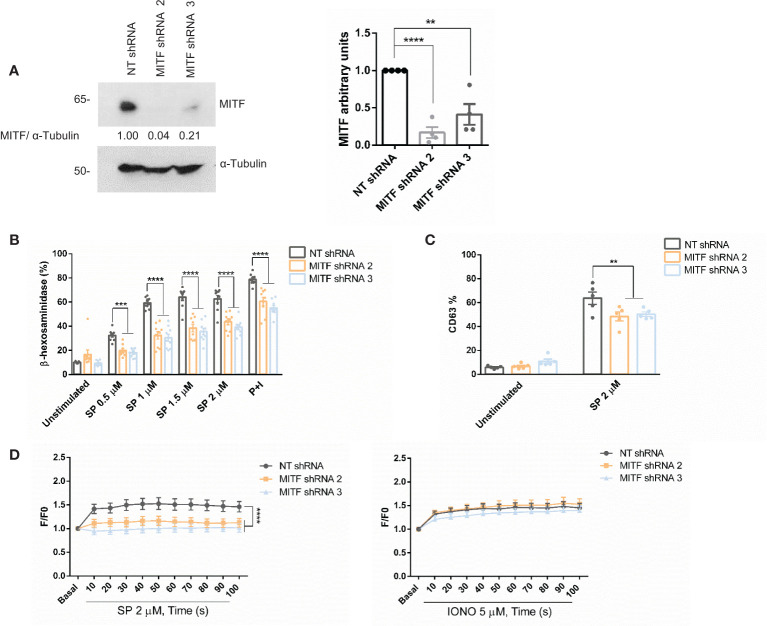
MITF silencing reduces degranulation and Ca2^+^ influx. **(A)** LAD2 cells were transduced with control NT (non-target) shRNA or MITF shRNA 2 or MITF shRNA 3, and the efficiency of MITF silencing was assessed by western blot. α-Tubulin was used as a loading control. The unpaired t-test was used for statistical analysis (**p < 0.01*, ****p < 0.0001). Experiments are the mean ± SEM (n=4). **(B)** β-hexosaminidase was performed with different concentrations of SP and P+I (PMA+ionomycin, 10 ng/mL+0.5 μM) as a positive control in NT and MITF-silenced cells, n=4. **(C)** Degranulation was determined by flow cytometry using CD63 staining in unstimulated and SP (2uM) in the alive population (propidium iodide negative staining) in NT and MITF-silenced cells. **(D)** NT and MITF-silenced LAD2 cells were preincubated with Fluo4 and activated with SP (2µM) or ionomycin. A two-way ANOVA test was used for statistical analysis (**p<0.01, ***p<0.001, ****p<0.0001). Experiments are the mean ± SEM (n=3).

We next explored the role of MITF in MRGPRX2-dependent degranulation. Cells were silenced using lentivirus technology with two specific sequences to that end. The two sequences satisfactorily knocked down the levels of MITF (**Figure 4A**). Degranulation measured by β-hexosaminidase release (**Figure 4B**) and CD63 expression by FACS (**Figure 4C** and **Supplementary Figure 1A**) showed a significant reduction after SP stimulation. In addition, calcium influx significantly diminished after MITF silencing in MRGPRX2 activation (**Figure 4D**). None or little difference was observed when ionomycin was used as a primary stimulus. MRGPRX2 expression levels after MITF silencing were consistent, and all cells were positive (**Supplementary Figure 2A**). The mean of fluorescence intensity was lower, however, total MRGPRX2 levels analyzed by blotting showed no reduction in MITF silencing samples compared to Non-target (NT) control (**Supplementary Figure 3**). MITF is involved in cell survival in different cellular models (46, 47), thus downregulation of this transcription factor may induce apoptosis. Next, we assessed apoptosis in MITF-knockdown cells. MITF silencing significantly increased cellular apoptosis (**Supplementary Figure 4A**). The increase in apoptosis was higher in cells with lower MITF levels. An equal number of live cells were used in all assays, exclusion of dead cells was performed when possible and MRGPRX2-dependent degranulation and calcium influx were still significantly decreased, in MITF-silenced cells.

### ML329 inhibits MITF expression and impairs MRGPRX2-dependent mast cell degranulation

**Figure 5:**
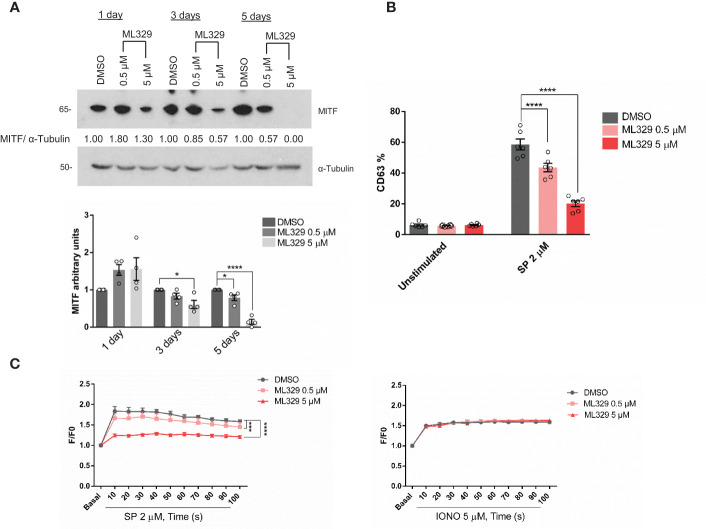
The MITF inhibitor ML329, reduces degranulation and Ca2^+^ influx. **(A)** LAD2 cells were treated with either DMSO or ML329 at different concentrations on various days, and the efficiency of MITF silencing was assessed by western blot. α-Tubulin was used as a loading control. The unpaired t-test was used for statistical analysis after 5 days (*p < 0.05, ****p<0.0001). Experiments are the mean ± SEM (n=4). **(B)** Degranulation was determined by flow cytometry using CD63 staining in unstimulated and ML329 -treated cells (after 5 days) in the live population (propidium iodide negative staining). **(C)** DMSO and ML329 -treated cells (after 5 days) were preincubated with Fluo4 and activated with SP (2µM) or ionomycin. A two-way ANOVA test was used for statistical analysis (***p<0.001, ****p<0.0001). Experiments are the mean ± SEM (n=3).

ML329 has been described to inhibit the MITF pathway (38, 48) therefore we used this inhibitor to reinforce data about MITF involvement in MRGPRX2 signaling. Cells treated with different concentrations of ML329 showed a decrease in MITF expression (**Figure 5A**). MITF reduction was always more consistent after day 5 thus the following experiments were performed on that day. Cells treated with ML329 showed decreased degranulation measured by CD63 expression (**Figure 5B** and **Supplementary Figure 1B**). Calcium influx was also impaired after MRGPRX2 activation, although the ionomycin response was not significantly affected (**Figure 5C**). MRGPRX2 expression after ML329 was similar in all cases (**Supplementary Figure 2B**). Like MITF silencing, ML329 also induced significant cellular apoptosis at a higher concentration (**Supplementary Figure 4B**). Altogether, decreased MITF levels correlated with reduced MRGPRX2-dependent degranulation and calcium influx.

**Figure 6:**
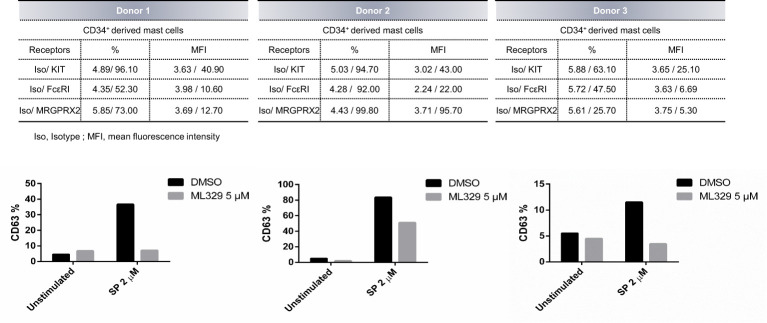
MITF inhibitor ML329 reduces CD34^+^ derived hMCS degranulation. CD34^+^ derived hMCs from three different healthy donors were assessed. KIT, FcϵRI, and MRGPRX2 were checked by flow cytometry. Degranulation was determined by flow cytometry using CD63 staining in unstimulated and ML329-treated cells (after 5 days) in the live population (propidium iodide negative staining).

CD34^+^ -derived human MCs were also used to analyze the effect of MITF reduction on degranulation. Human MCs fully differentiated from CD34 cells were treated with ML329 for five days. Cells were activated with SP, and CD63 staining was used to measure degranulation (**Figure 6**). The analysis was performed in the live cell population (propidium iodide negative). Our results show that ML329 treatment led to a significant reduction in MRGPRX2-dependent mast cell degranulation.

### Drug activation of MRGPRX2 induces MITF activity

One of the fascinating biological aspects that makes this receptor a current hot research topic is its ability to interact with several drugs and be involved in adverse drug reactions.

**Figure 7:**
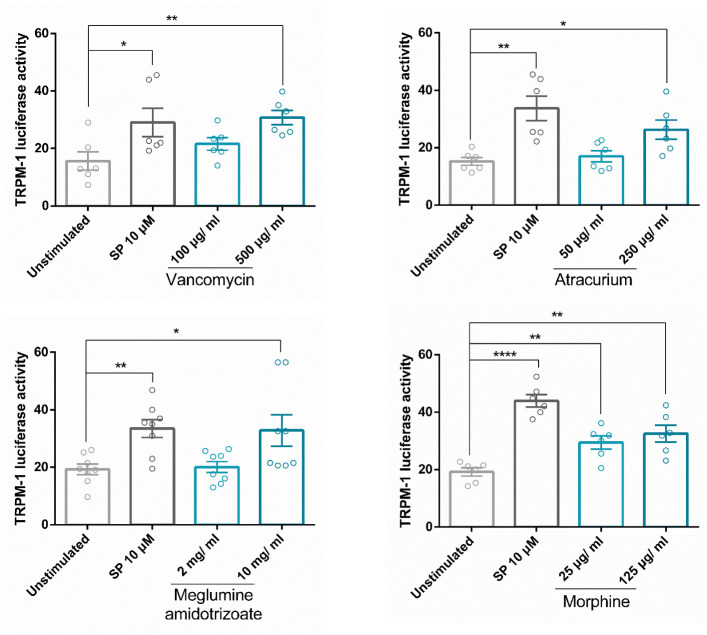
Drug activation of MRGPRX2 induces MITF activity. Cells were activated with SP or drugs (vancomycin, atracurium, morphine, and meglumine amidotrizoate) reported to induce MRGPRX2-dependent MC degranulation at various concentrations for 6 hours in LAD2 cells transfected with TRPM1-luciferase gene reporter. Experiments are the mean ± SEM (n=3 for vancomycin and atracurium, n=4 for meglumine amidotrixoate and morphine). The unpaired t-test was used for statistical analysis (*p < 0.05, **p < 0.01, ****p < 0.0001).

Our group showed that vancomycin, atracurium, and morphine induce MRGPRX2-dependent degranulation (8). Meglumine amidotrizoate was also described to interact with MRGPRX2 (49). Next, we analyzed whether these drugs were able to increase MITF activity by using Melastatin 1 (TRPM1) promoter-controlled firefly luciferase. Our results show that all were able to increase MITF activity (**Figure 7**). These data indicate that MITF activity is induced by several drugs reported to mediate their actions through MRGPRX2 signaling thus MITF is a MRGPRX2-dependent downstream signal for endogenous (SP) and exogenous ligands (atracurium, vancomycin…).

## Discussion

Despite the increasing number of studies on MRGPRX2 in recent years, the dissection of its signaling pathway in MCs is poorly understood. Several studies focus on identifying new receptor ligands and how to block ligand recognition or involvement in pathology. To advance in the knowledge of MRGPRX2, one significant limitation is the lack of the crystal structure which prevents a complete characterization of ligand binding sites and a proper design of agonists/antagonists. Nevertheless, molecular modeling experiments have identified crucial amino acids for endogenous binding ligands, such as substance P, and exogenous ligands such as cationic agonists or opioids (12, 50, 51). MRGPRX2-triggered degranulation depends on Gαi, Gαq, Ca^++^ channels, ERK, and PI3K (14, 15). Cytoskeleton dynamics and granule exocytosis need Cdc42 GTPase activation and the participation of unconventional class I myosins, MYO1F (16), and β1 and β2 arrestins regulate the kinetics and the extent of MRGPRX2 cellular activity (17). The present study adds more insights into MRGPRX2 signals and demonstrates the involvement of the LysRS-MITF pathway.

LysRS is a moonlighting protein that belongs to the aminoacyl-tRNA synthetases (aaRS), which catalyze the aminoacylation reaction linking amino acids to their cognate tRNAs. They are highly conserved cytoplasmic and mitochondrial enzymes, one for each amino acid, and are responsible for the fidelity of gene code reading. During evolution, some aaRS acquired newly evolved domains that are not crucial for tRNA aminoacylation but are responsible for non-canonical functions (52). In this respect, LysRS, the aaRS that attaches lysine to its corresponding tRNA, is also involved in IgE-dependent signals upstream of the microphthalmia-associated transcription factor (MITF) in MCs (23). Upon IgE crosslinking, LysRS is phosphorylated (Ser 207) and translocated to the nucleus, where it catalyzes the synthesis of Ap4A as non-canonical activity. Ap4A binds to HINT, releasing MITF from the MITF-HINT1 inhibitory complex and causing an increase in the transcription of its target genes (23). MITF is a crucial hub in MC biology and function, according to the *Mitf*-mutant mouse phenotype, characterized by a reduced number and abnormal MCs (53–55).

In this study, we found that MRGPRX2 increases ERK1/2 phosphorylation rapidly and transiently. Our results agree with data showing that ERK1/2 is efficiently phosphorylated after MRGPRX2 in skin MCs and is more transiently induced than FcεRI signals (15). Nevertheless, this rapid signal allowed increased LysRS recruitment in the nucleus of MRGPRX2-activated cells. LysRS in the nucleus performs its non-canonical function, releasing MITF from the inactive HINT1-MITF complex (23). Moreover, MRGPRX2 signaling increased MITF phosphorylation (Ser 73). The transcriptional activity of MITF is primarily regulated post-translationally. Most studies relating to MITF regulation have been performed in melanocytes showing that mainly ERK1/2 phosphorylates MITF at Ser73, p90 ribosomal S6 kinase (p90RSK) at Ser409, glycogen synthase kinase-3β (GSK3β) at Ser298, and p38 MAPK at Ser307 (27–29). MITF phosphorylation usually augments its activity. MAPK-mediated phosphorylation of MITF at Serine 73 combines the two coactivators, E1A binding protein p300 and cAMP-response element binding protein (p300/CBP), enhancing MITF transcriptional activity (56). However, dual phosphorylation at Ser73 and Ser409 endorses its ubiquitination and proteasome degradation (29). MITF A is the longest and most abundant isoform in mast cells (57). Ser 73 in MITF-M, the specific isoform in melanocytes, corresponds to Ser 180 in MITF-A.

In our study, MRGPRX2 activation with SP increased MITF activity measured by the TRPM1-reporter gene. Overexpression of LysRS greatly enhanced MITF activity after SP stimulation. PGD2 production depends on MITF activity (33), thus, we observed that the augmented MITF activity was associated with an increase in PGD2. Altogether, MRGPRX2 ligation with substance P led to LysRS translocation to the nucleus, raising MITF activity. In parallel, MRGPRX2 signaling induced MITF phosphorylation, contributing to the increase in MITF activity.

MITF phosphorylation has been described as a mechanism of nuclear export-import of MITF to regulate the activity of this transcription factor in melanocytes (43). Interestingly, we found increased MITF in the cytosol after MRGPRX2 activation. Thus, it would be plausible that similar regulation events could occur in MCs. Nevertheless, this subject deserves further study.

MRGPRX2 ligation to exogenous ligands that can result in adverse drug reactions such as to atracurium, vancomycin, morphine, and meglumine amidotrizoate increases MITF activity, suggesting that endogenous and exogenous ligands use the LysRS-MITF pathway.

Selective silencing with MITF was effective after day 5, and previous experiments showed that the half-life of MITF was longer than expected for a transcription factor. Cycloheximide experiments from our group confirmed a complete reduction of protein levels after 96h drug treatment (data not shown). Similarly, using ML329, we see a significant decrease in protein levels at day 5. Our study indicates that MITF downregulation by shRNA silencing or ML329 treatment results in decreased MRGPRX2-dependent degranulation measured by β-hexosaminidase and CD63 plasma membrane expression. MITF function has been related to the biogenesis of lysosomes (58), and bone marrow-derived MCs from *Mitf* -/- mice display hypogranularity that can be restored with MITF addition (59). We examined whether our MITF-silenced cells had reduced granular content compared to control cells. Unfortunately, we could not find any apparent differences in our staining with Lysotracker in MITF-silenced cells compared to non-target controls (data not shown). Interestingly, downregulated MITF levels significantly decreased Ca2+ influx after substance P activation. MRGPRX2-dependent Ca2^+^ is regulated by store-operated Ca2^+^ entry (SOCE) *via* the calcium sensor, stromal interaction molecule 1 (STIM1) (60). STIM1 is an endoplasmic reticulum (ER) Ca2^+^ sensor that, upon activation and decreased ER Ca2^+^ levels, oligomerizes and activates Orai channels (Orai1/2/3), resulting in Ca2^+^ influx (61). Indeed, STIM1 is crucial in promoting the Ca2^+^ influx needed for IgE-dependent mast cell activation and anaphylaxis (62). Recently, MITF has been reported to regulate STIM1 and SOCE expression in melanocytes (63). Chromatin immunoprecipitation (ChIP) and luciferase assays with truncated STIM1 promoters validated the MITF-STIM1 interaction. Functional assays confirmed that MITF regulates STIM1 expression and activity in primary human melanocytes (63). Further experiments are needed to see MITF-dependent STIM1 regulation in MCs.

A pool of MITF resides in mitochondria and regulates proteins independently of its function as a transcription factor of nuclear genes. MITF interacts with one of the three subunits of the pyruvate dehydrogenase (PDH) complex, an enzyme that catalyzes the conversion of pyruvate to acetyl CoA (64). Moreover, mitochondrial MITF regulates PDH activity, which is crucial to maintain glucose homeostasis and essential as a source of mitochondrial ATP to maintain energetic expenses for MC degranulation and cytokine secretion. MITF activation related to the phosphorylation of Serine 73 mediated by ERK1/2 activity accounts for increased mast cell activity (65).

MITF downstream of the KIT receptor governs mast cell differentiation and proliferation (66). KIT receptor signaling regulates MITF through miR-539 and miR381 downregulation (22). At the same time, MITF fosters KIT expression (67), showing a reciprocal regulation. The reduced and abnormal MC in *Mitf*-mutated mice is partly due to the low levels of KIT receptor and, hence, the low response to its ligand, SCF (68). In that context, our group reported that the adaptor protein 3BP2 participates in the KIT-MITF axis, delivering survival signals in MCs. Thus, silencing of 3BP2 reduced KIT and MITF protein levels and induced MC apoptosis. MITF overexpression rescued KIT protein levels in 3BP2 knockdown cells (69).

Since a reduction in MITF may affect cell viability, apoptosis was monitored. Indeed, MITF downregulation is also involved in the termination of MC-mediated response, apoptosis induction, and removal of exhausted cells (70, 71). Conversely, increased MITF levels and activity have been linked to increased mast cell activity and proliferation. MITF is required for the transformed phenotype of mastocytosis (72). Indeed, MITF downregulation leads to apoptosis of HMC-1 carrying the gain of function mutation KIT D816V (73). Moreover, MITF is highly expressed in bone marrow biopsies from patients with systemic mastocytosis and activating KIT mutations (22). Recently, we identified that a mutation in LysRS (P542R) in a patient with severe anaphylaxis to wasp venom favors the recruitment of this molecule to the nucleus resulting in a constitutive increase in MITF activity (34). This increased MITF activity accounts for an increase in histamine and PGD2 secretion. It is known that transcription factors GATA2 and MITF regulate Hdc gene expression, which is necessary for IgE/mast cell-mediated anaphylaxis (31).

Altogether, the LysRS-MITF pathway should be considered in MRGPRX2 signaling, and it is shared with FcεRI. Patients with alterations in this pathway may increase their range of susceptibility to a broad spectrum of substances that can trigger both receptors.

Increased knowledge of MRGPRX2 signaling may provide new approaches for upregulating responses, which may help treat antibiotic-resistant cutaneous infections, or downregulate MRGPRX2 to ameliorate allergic and inflammatory diseases *via* this receptor.

## Data availability statement

The original contributions presented in the study are included in the article/**Supplementary Material**. Further inquiries can be directed to the corresponding author.

## Ethics statement

The studies involving human participants were reviewed and approved by University of Barcelona. Written informed consent for participation was not required for this study in accordance with the national legislation and the institutional requirements.

## Author contributions

YG performed and designed the experiments and wrote and reviewed the manuscript. LO, EP-P, and CA performed the experiments and reviewed the manuscript. MG provided technical support and reviewed the manuscript. RM-C conceived the experiments and reviewed the manuscript. MM conceived the experiments, provided secure funding, and wrote and reviewed the manuscript. All authors contributed to the article and approved the submitted version.
