# Supplementary material for: MRGPRX2 signaling involves the Lysyl-tRNA synthetase and MITF pathway
Source: Front Immunol. 2023 May 10;14:1154108. doi: 10.3389/fimmu.2023.1154108 (PMC10206166; doi:10.3389/fimmu.2023.1154108)
Supplement: Supplementary file 1 [file Image_1.pdf]

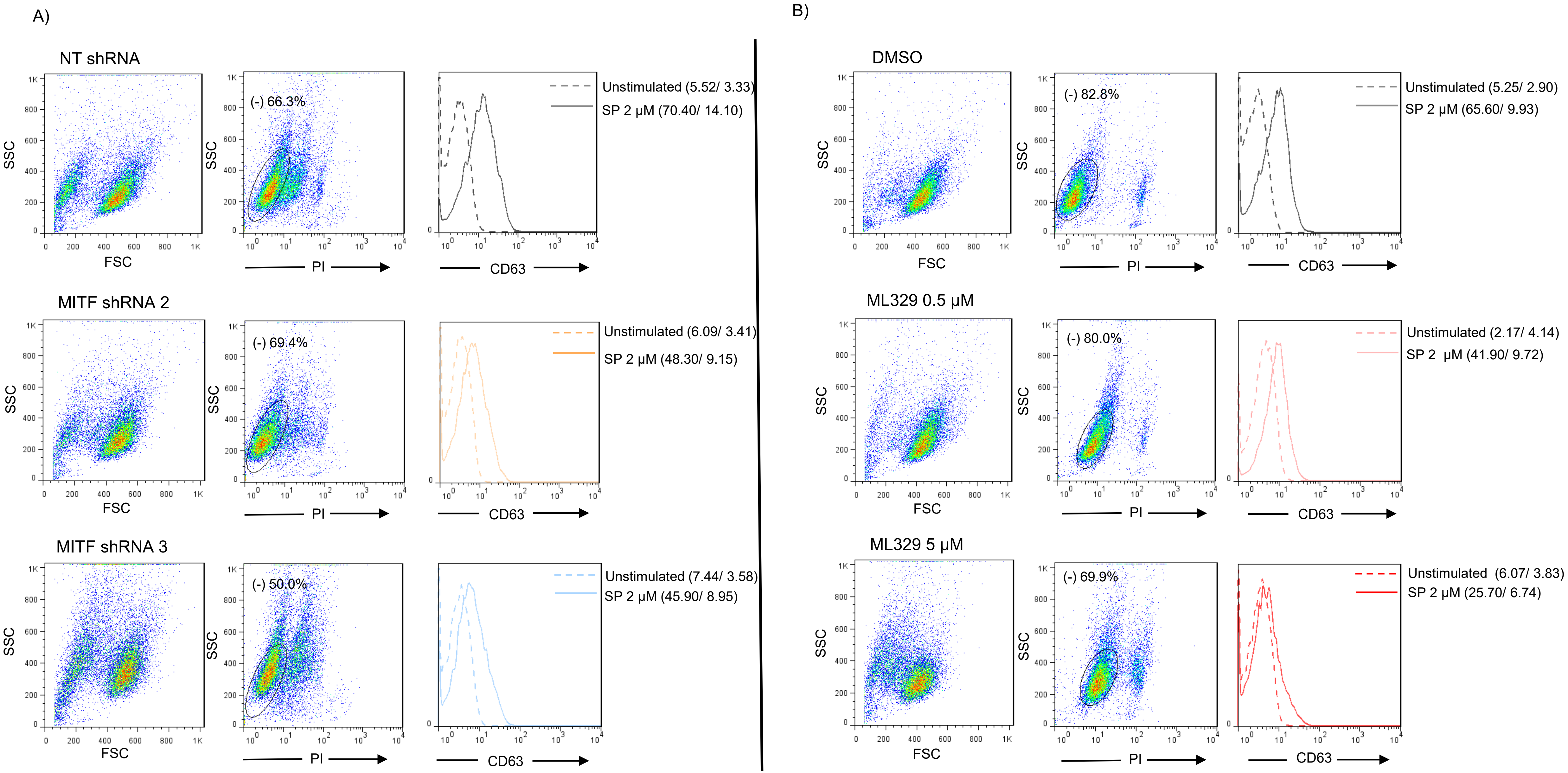

Supplementary Figure 1. CD63 expression after MITF silencing or ML329 treatment in propidium iodide (PI) negative gated LAD2 cells. (A) CD63 expression was measured in NT shRNA, MITF shRNA 2, and MITF shRNA 3 transduced cells, after five days of infection, by flow cytometry; and (B) after DMSO or 0.5 µM or 5µM ML329 treatment for five days. The (%/ MFI) of CD63 was shown for unstimulated and 2 µM SP stimulated cells in the same plot. MFI: Mean of Fluorescence Intensity. Notice that ML329 has some intrinsic fluorescence.
