# Supplementary material for: MRGPRX2 signaling involves the Lysyl-tRNA synthetase and MITF pathway
Source: Front Immunol. 2023 May 10;14:1154108. doi: 10.3389/fimmu.2023.1154108 (PMC10206166; doi:10.3389/fimmu.2023.1154108)
Supplement: Supplementary file 2 [file Image_2.pdf]

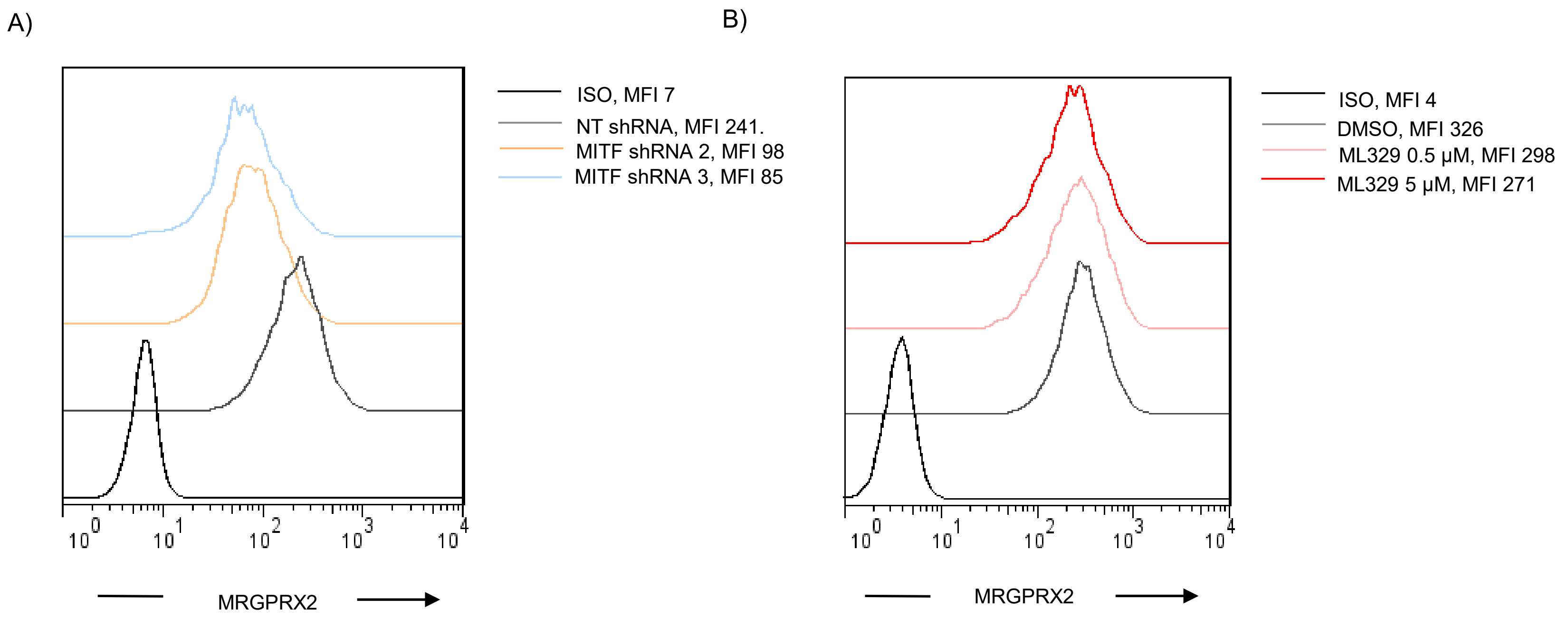


**Supplementary Figure 2. MRGPRX2 levels after MITF silencing or ML329 treatment in LAD2 cells.** (A) MRGPRX2 expression was measured in NT shRNA, MITF shRNA 2, and MITF shRNA 3 transduced cells (day five after infection) by flow cytometry. (B) MRGPRX2 expression was measured in cells incubated with DMSO, 0.5 μM ML329, or 5 μM ML329 for five days by flow cytometry. MFI: Mean of Fluorescence Intensity.
