# Supplementary material for: MRGPRX2 signaling involves the Lysyl-tRNA synthetase and MITF pathway
Source: Front Immunol. 2023 May 10;14:1154108. doi: 10.3389/fimmu.2023.1154108 (PMC10206166; doi:10.3389/fimmu.2023.1154108)
Supplement: Supplementary file 3 [file Image_3.pdf]

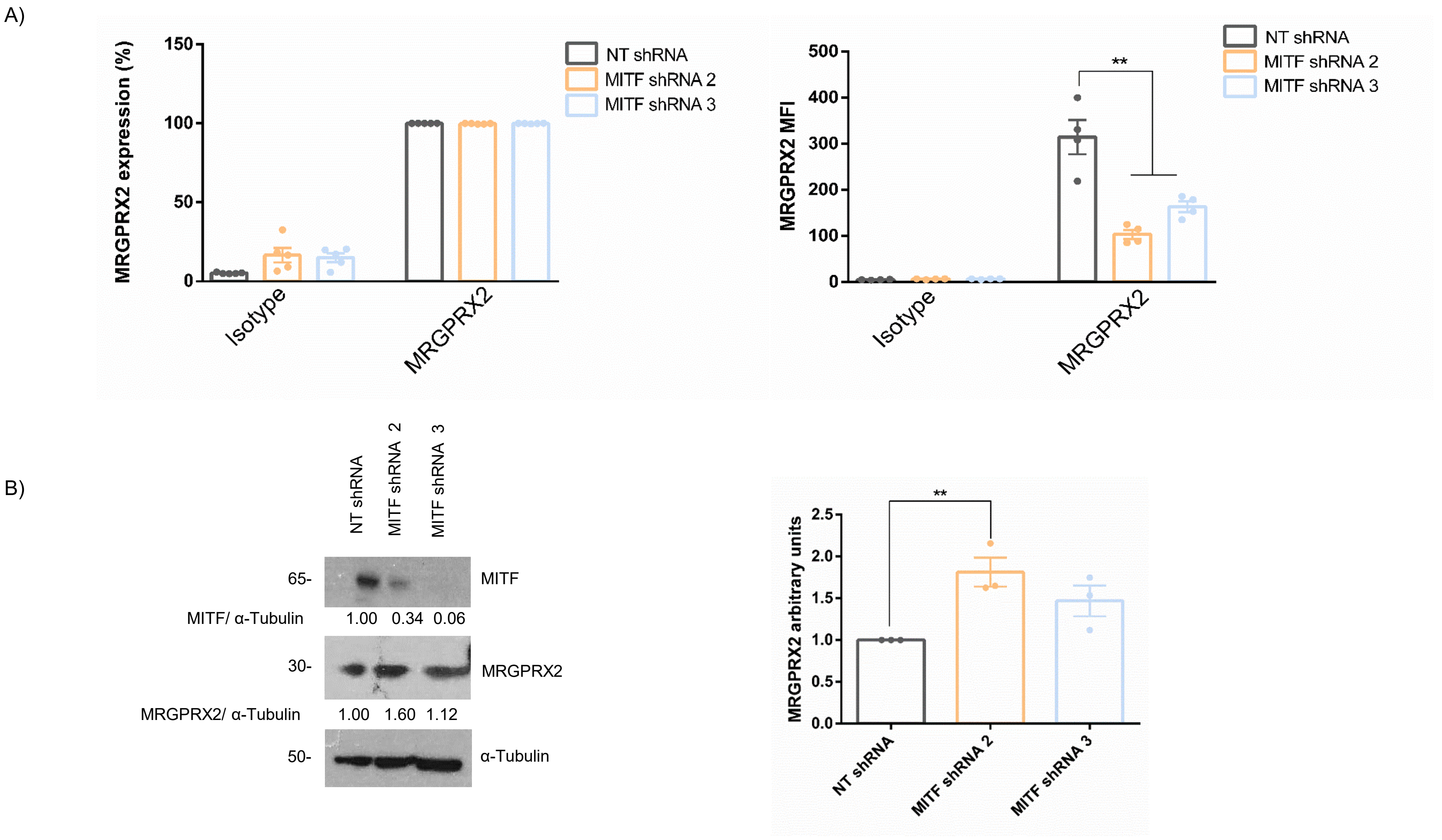

Supplementary Figure 3. MRGPRX2 expression after MITF silencing. (A) MRGPRX2 expression (% and Mean of Fluorescence Intensity (MFI) was measured by flow cytometry (n=4). (B) Total MRGPRX2 levels were measured by western blot (n=3). The unpaired t-test was used to determine significant differences, ** $p < 0.01$.
