# Supplementary material for: MRGPRX2 signaling involves the Lysyl-tRNA synthetase and MITF pathway
Source: Front Immunol. 2023 May 10;14:1154108. doi: 10.3389/fimmu.2023.1154108 (PMC10206166; doi:10.3389/fimmu.2023.1154108)
Supplement: Supplementary file 4 [file Image_4.pdf]

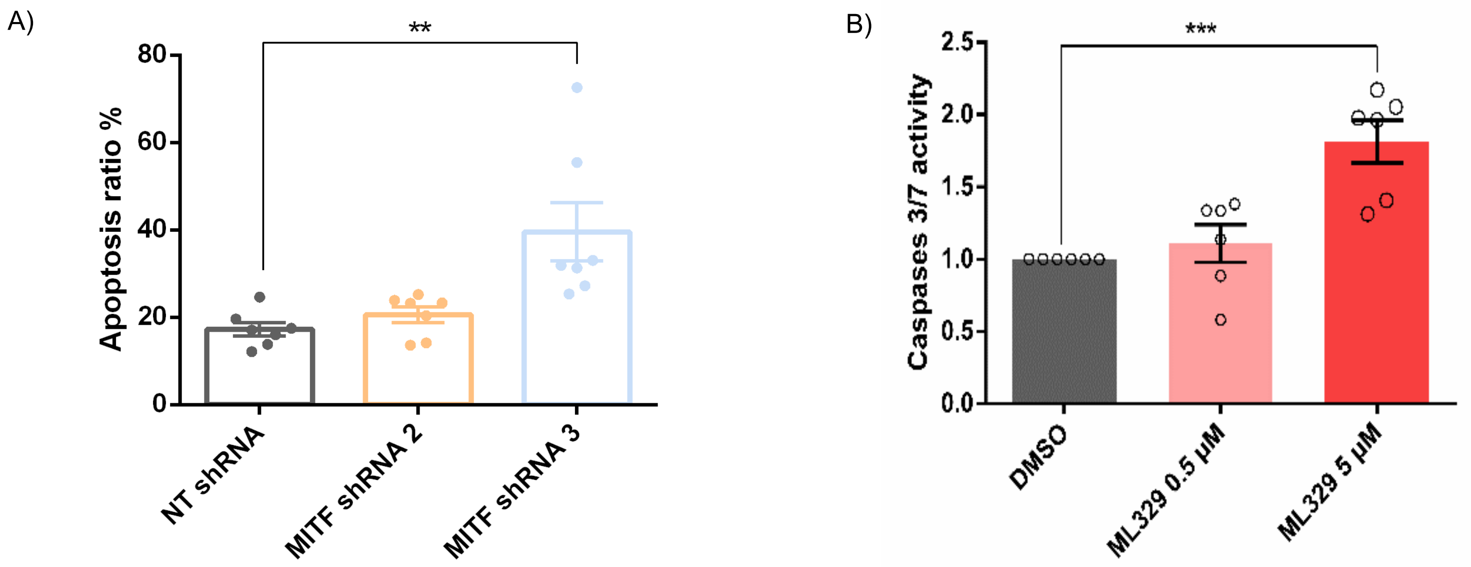

Supplementary Figure 4. MITF knockdown increases apoptosis in mast cells. (A) Apoptosis assays (Annexin %) in NT shRNA, MITF shRNA 2 and MITF shRNA 3 transduced LAD2 cells were assessed. (B) Caspase 3/7 activity was measured after incubating LAD 2 cells with DMSO, or ML329 (day 5). The unpaired t-test was used for statistical analysis (**$p<0.01$, ***$p<0.001$). Experiments are the mean ±SEM (n=3).
