# Supplementary material for: MRGPRX2 signaling involves the Lysyl-tRNA synthetase and MITF pathway
Source: Front Immunol. 2023 May 10;14:1154108. doi: 10.3389/fimmu.2023.1154108 (PMC10206166; doi:10.3389/fimmu.2023.1154108)
Supplement: Supplementary file 5 [file DataSheet_1.docx]

Figure 1

Figure 1

WB: а-LysRS

Cytoplasm Nucleus





0’,5’,15’

0’,5’,15’


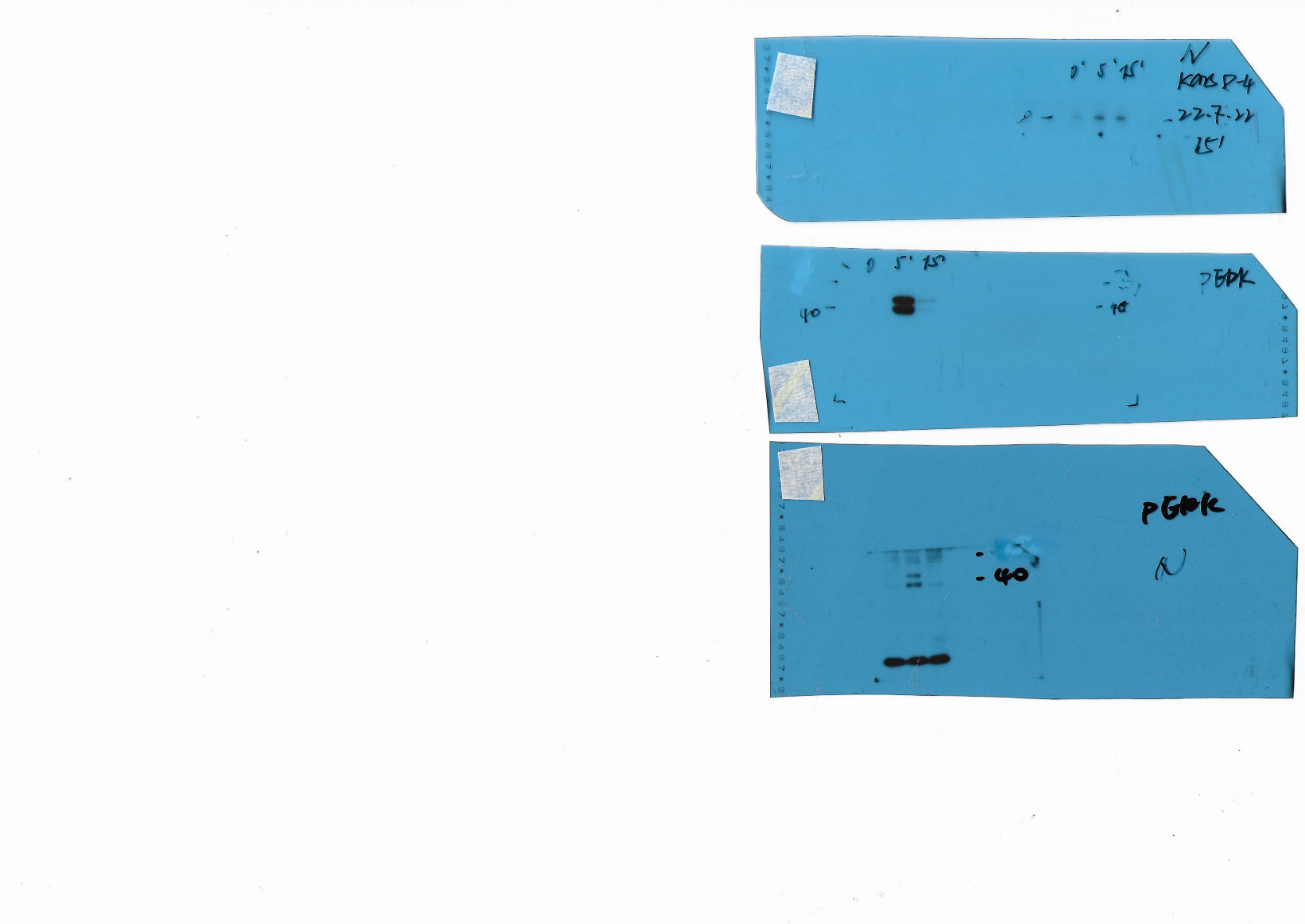


WB: α-LysRS

WB: pERK

WB: а-LysRS


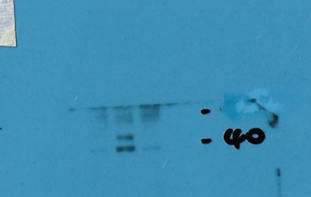

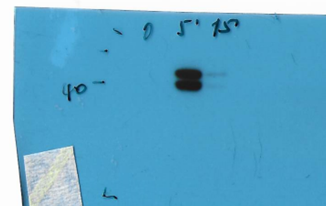


WB: pERK

WB: а-Tubulin


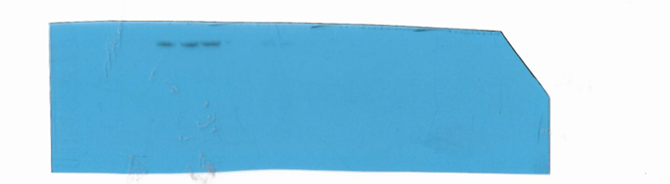


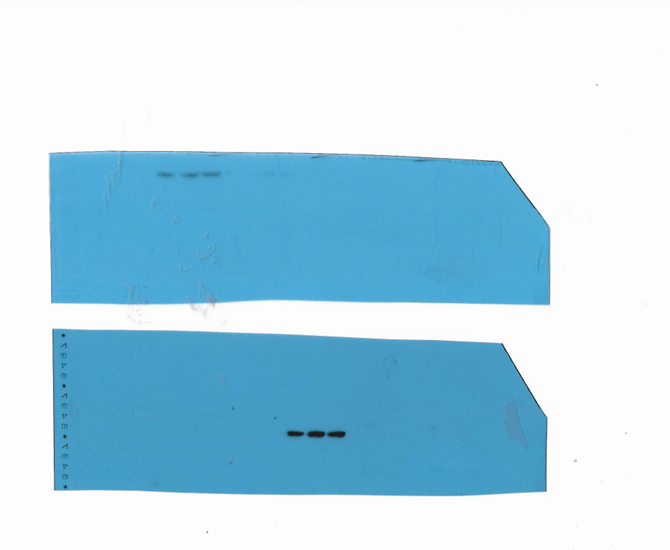


WB: α-Tubulin

WB: а-Laminin B1


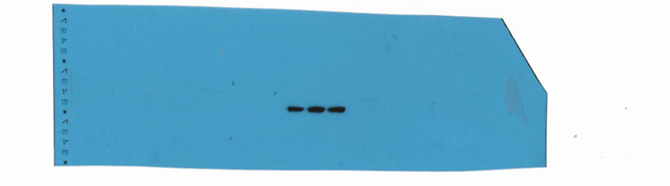

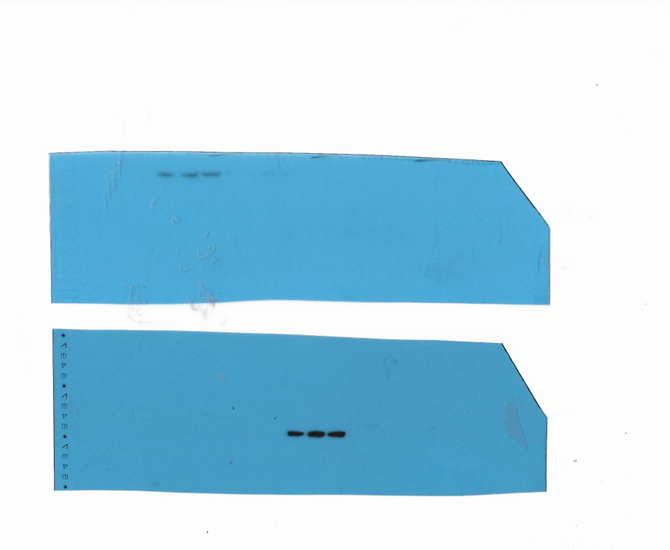


WB: α-Laminin B1

Cytoplasm Nucleus

0’,5’,15’

0’,5’,15’

Figure 2


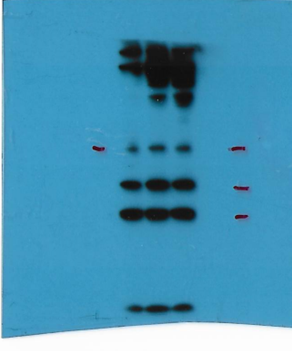

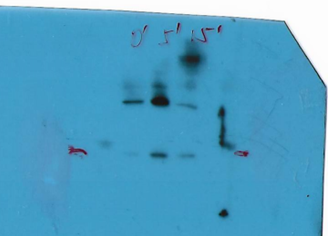


WB: pMITF




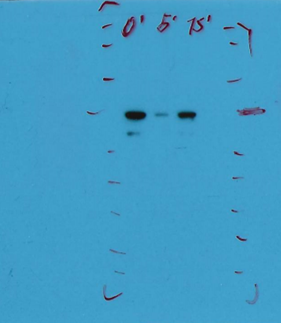

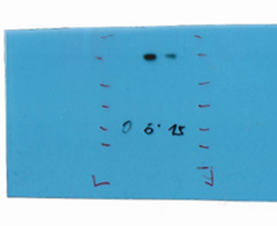


WB: MITF


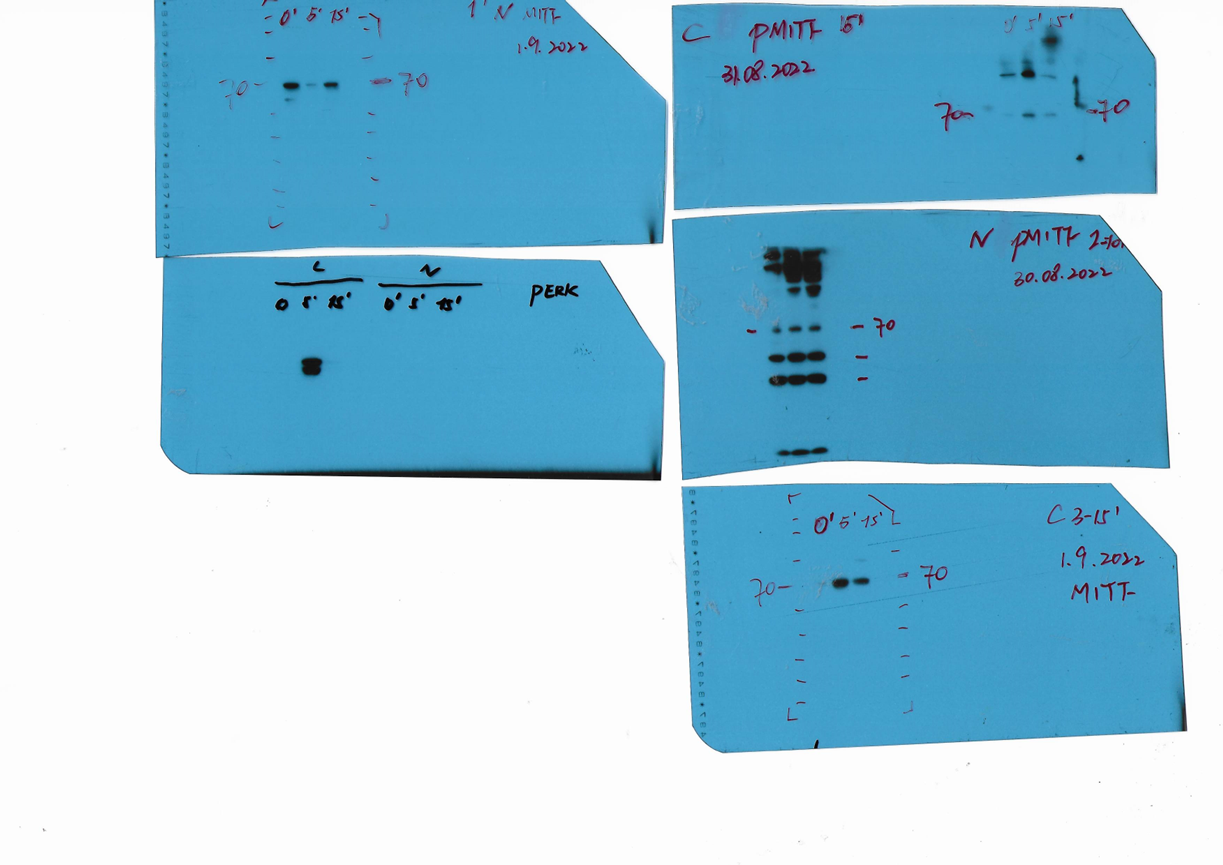


WB: pERK


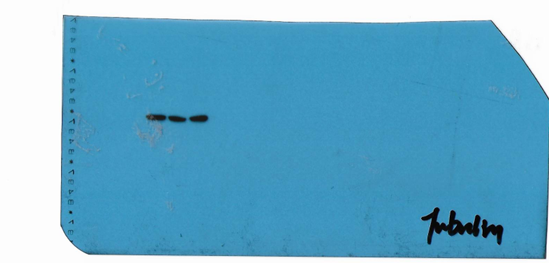




WB: α-Tubulin


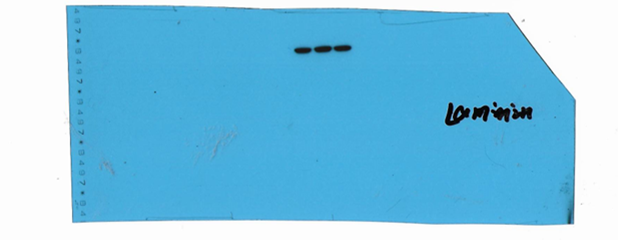

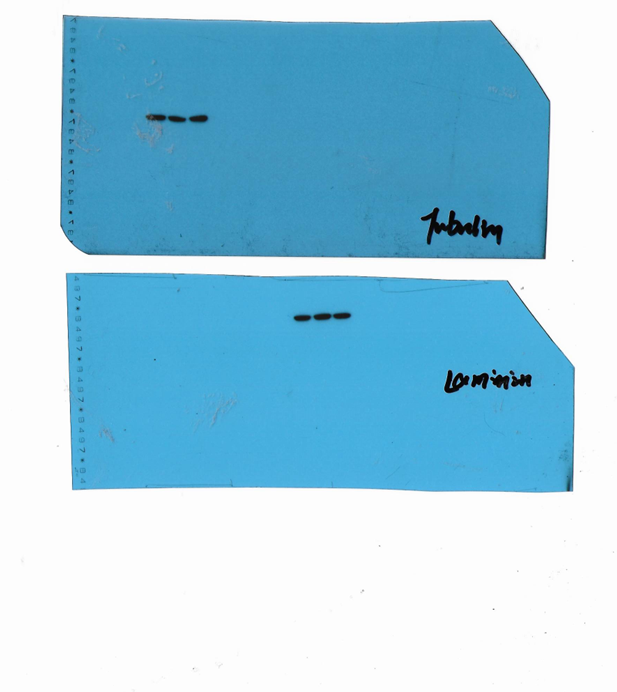


WB: α-Laminin B1

Figure 3

ShRNAMITF 3

ShRNAMITF 2

NT


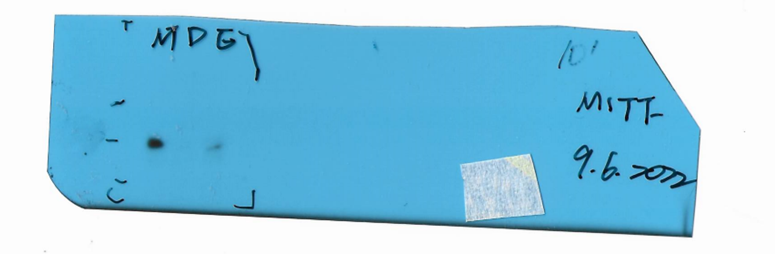


WB: MITF


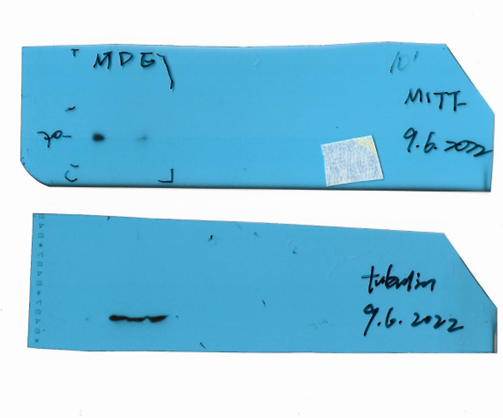


WB: α-Tubulin

Figure 4

1 day 3 days 5 days

ML329 5 uM

ML329 0.5 uM

DMSO

ML329 5 uM

ML329 0.5 uM

DMSO

ML329 5 uM

ML329 0.5 uM

DMSO


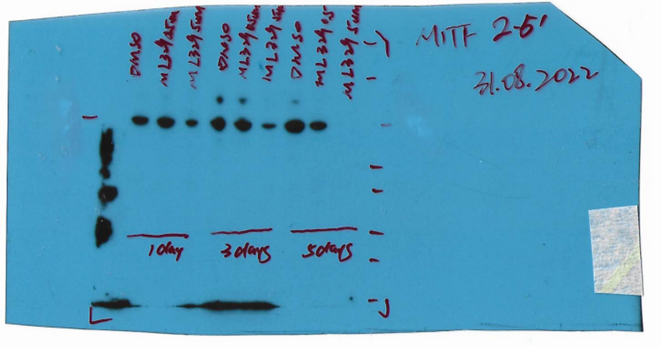


WB: MITF


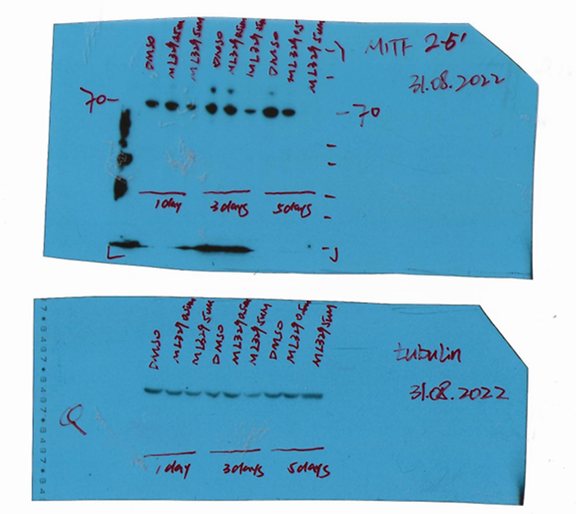


WB: α-Tubulin

Suppl Figure 3

ShRNAMITF 3

ShRNAMITF 2

NT


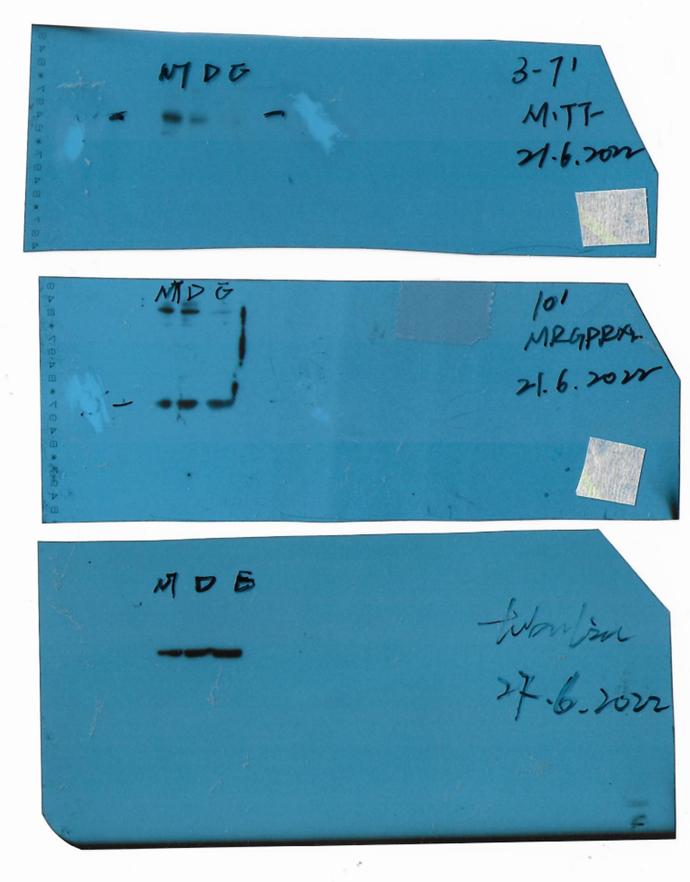


WB: MRGPRX2

WB: α-Tubulin

WB: MITF
